# Increased Magnetic Susceptibility in the Deep Gray Matter Nuclei of Wilson's Disease: Have We Been Ignoring Atrophy?

**DOI:** 10.3389/fnins.2022.794375

**Published:** 2022-06-01

**Authors:** Xiao-Zhong Jing, Xiang-Zhen Yuan, Gai-Ying Li, Jia-Lin Chen, Rong Wu, Ling-Li Yang, Shu-Yun Zhang, Xiao-Ping Wang, Jian-Qi Li

**Affiliations:** ^1^Department of Neurology, Tongren Hospital, Shanghai Jiao Tong University School of Medicine, Shanghai, China; ^2^Department of Neurology, Weifang People's Hospital, Weifang, China; ^3^Shanghai Key Laboratory of Magnetic Resonance, School of Physics and Electronic Science, East China Normal University, Shanghai, China; ^4^Department of Neurology, Jiading Branch of Shanghai General Hospital, Shanghai Jiao Tong University School of Medicine, Shanghai, China

**Keywords:** Wilson's disease, quantitative susceptibility mapping, total metal content, brain atrophy, mean bulk susceptibility, diagnosis

## Abstract

**Background:**

Histopathological studies in Wilson's disease (WD) have revealed increased copper and iron concentrations in the deep gray matter nuclei. However, the commonly used mean bulk susceptibility only reflects the regional metal concentration rather than the total metal content, and regional atrophy may affect the assessment of mean bulk susceptibility. Our study aimed to quantitatively assess the changes of metal concentration and total metal content in deep gray matter nuclei by quantitative susceptibility mapping to distinguish patients with neurological and hepatic WD from healthy controls.

**Methods:**

Quantitative susceptibility maps were obtained from 20 patients with neurological WD, 10 patients with hepatic WD, and 25 healthy controls on a 3T magnetic resonance imaging system. Mean bulk susceptibility, volumes, and total susceptibility of deep gray matter nuclei in different groups were compared using a linear regression model. The area under the curve (AUC) was calculated by receiver characteristic curve to analyze the diagnostic capability of mean bulk susceptibility and total susceptibility.

**Results:**

Mean bulk susceptibility and total susceptibility of multiple deep gray matter nuclei in patients with WD were higher than those in healthy controls. Compared with patients with hepatic WD, patients with neurological WD had higher mean bulk susceptibility but similar total susceptibility in the head of the caudate nuclei, globus pallidus, and putamen. Mean bulk susceptibility of putamen demonstrated the best diagnostic capability for patients with neurological WD, the AUC was 1, and the sensitivity and specificity were all equal to 1. Total susceptibility of pontine tegmentum was most significant for the diagnosis of patients with hepatic WD, the AUC was 0.848, and the sensitivity and specificity were 0.7 and 0.96, respectively.

**Conclusion:**

Brain atrophy may affect the assessment of mean bulk susceptibility in the deep gray matter nuclei of patients with WD, and total susceptibility should be an additional metric for total metal content assessment. Mean bulk susceptibility and total susceptibility of deep gray matter nuclei may be helpful for the early diagnosis of WD.

## Introduction

Wilson's disease (WD) is an autosomal-recessive disorder of copper metabolism. The causative gene, *ATP7B*, encodes a copper-transporting P-type ATPase ATP7B, which plays an important role in maintaining copper homeostasis and the synthesis of functional ceruloplasmin (Bandmann et al., 2015; Wang and Wang, 2019). The dysfunction of ATP7B leads to pathological accumulation of copper in the liver and brain, causing liver disease and neurological dysfunction. Liver disease presents as the first clinical symptom in 40–60% of patients with WD (Kozic et al., 2003; Czlonkowska et al., 2018). Although neurological symptoms generally occur between the ages of 20 and 30, about 10 years after the onset of hepatic symptoms (Lorincz, 2010), children may also manifest neurological symptoms. According to the presence or absence of neurological symptoms, WD can be divided into neurological form of WD (neuro-WD) and hepatic form of WD (hep-WD). It is assumed that high copper content in the brain leads to brain tissue damage and neurological dysfunction (Bruehlmeier et al., 2000; Dusek et al., 2015, 2019; Czlonkowska et al., 2018).

Studies using traditional GRE MRI sequences such as T2/T2*-weighted images and susceptibility-weighted images (SWI) have identified metal accumulation in the brains of patients with WD (Skowronska et al., 2013). Although traditional T2^*^-weighted images and SWI are sensitive to paramagnetic metal accumulation, they suffer from blooming artifacts and the non-local effect. Most importantly, traditional GRE MRI sequences cannot quantify any susceptibility source without deconvolution (Wang et al., 2017). R2^*^ values have been used to measure iron deposition in brain nuclei. A recent histopathological study reported that copper and iron concentrations were significantly increased in the basal ganglia of patients with WD, and the R2^*^ values of basal ganglia were associated with iron concentration rather than copper concentration (Dusek et al., 2018). However, R2^*^ mapping cannot distinguish between iron and diamagnetic species and depends on factors such as field strength and homogeneity of iron distribution (Bilgic et al., 2012). Quantitative susceptibility mapping (QSM) is a postprocessing technique using the GRE phase images and solves the deconvolution problem from magnetic field to susceptibility source. The advantage of QSM is that it can measure the true susceptibility of tissue and is little affected by the external magnetic field (de Rochefort et al., 2010; Wang et al., 2017; An et al., 2018; Lee et al., 2018). Studies based on QSM found significantly increased susceptibility in the deep gray matter (DGM) nuclei and brain stem of patients with WD (Fritzsch et al., 2014; Doganay et al., 2018; Dezortova et al., 2020; Li et al., 2020; Yuan et al., 2020). It is worth noting that both R2^*^ and QSM have been recognized as measures of iron concentration.

Recently, a study revealed that DGM nuclei atrophy in multiple sclerosis patients can affect the assessment of mean R2^*^ values, and the increase in mean R2^*^ may be caused by regional atrophy rather than iron accumulation (Hernandez-Torres et al., 2019). The commonly utilized mean bulk susceptibility only reflects metal concentration, which not necessarily translates into total metal content. For example, mean bulk susceptibility will increase when the total metal content does not change while the volume of the DGM nuclei decreases. Brain atrophy in patients with WD has been repeatedly reported and the severity of brain atrophy may be associated with neurological and functional impairments in patients with WD (Smolinski et al., 2019; Zou et al., 2019). A rating scale for semiquantitative assessment for the severity of WD using brain MRI has been developed and validated (Dusek et al., 2020). The severity of neurological symptoms was mainly related to the chronic damage score, especially the severity of brain atrophy (Dusek et al., 2020). Dusek et al. recently used deformation and surface-based morphometry found that WD caused widespread brain atrophy, which were most pronounced in DGM nuclei, the primary motor, premotor and visual cortices, as well as in white matter within the pons, mesencephalon, internal capsule, and adjacent lobes (Dusek et al., 2021). The putamen (Put) volume was associated with the Unified WD Rating Scale score and might be used as a surrogate imaging marker of clinical severity (Dusek et al., 2021). As brain atrophy is a common imaging feature of WD, we speculate that mean bulk susceptibility may not enough to reflect the increased metal accumulation in WD, and total susceptibility may be an additional metric for total metal content assessment account for the effects of tissue atrophy.

In this study, we hypothesized that DGM nuclei atrophy could affect the assessment of metal concentration, and total susceptibility may be an additional metric for total metal content assessment account for the effects of tissue atrophy. Both mean bulk susceptibility and total susceptibility of DGM nuclei may be useful for the diagnosis of WD. To test this hypothesis, we first investigated metal concentration and total metal content changes in the DGM nuclei of patients with WD by evaluating mean bulk susceptibility and total susceptibility using QSM, and the volumes of DGM nuclei were also calculated. Then, we assessed the capability of mean bulk susceptibility and total susceptibility in discriminating patients with WD from healthy controls (HCs).

## Materials and Methods

### Subjects

A total of 30 patients with WD were recruited from the Department of Neurology, Tongren Hospital, Shanghai Jiao Tong University School of Medicine, between December 2018 and December 2019. WD was diagnosed according to the Leipzig diagnostic scoring system, and the diagnosis was established if the total score ≥ 4 (Ferenci et al., 2003; European Association for Study of Liver, 2012). Neuro-WD was defined as the presence of neurological symptoms at the onset or any time during the course of the disease. Neurological symptoms of patients with WD were evaluated by two neurologists with consensus. Patients with WD without any neurological symptoms were defined as hep-WD. Exclusion criteria included severe neurological symptoms or severe liver decompensation, and history of other neuropsychiatric disorders or liver disease. All patients with WD were on de-copper treatment with D-penicillamine or dimercaptosuccinic acid (DMSA). A total of 25 HCs were recruited from our coworkers and college students who were age and gender matched. All the HCs had no history of neuropsychiatric or hepatic diseases. This study was approved by the Medical Ethics Committee of Tongren Hospital, Shanghai Jiao Tong University School of Medicine. Informed consent was obtained from all subjects before participation.

### Imaging Data Acquisition and Procession

All subjects were scanned on a clinical 3T MRI system (Magnetom Prisma Fit, Siemens Healthcare, Erlangen, Germany) equipped with a 20-channel head matrix coil. The susceptibility maps were acquired from a 3D spoiled multi-echo gradient-echo (GRE) sequence with the following imaging parameters, namely, repetition time (TR) = 31 ms, the first echo time (TE1) = 4.07 ms, echo spacing (ΔTE) = 4.35 ms, numbers of echoes = 6, flip angle = 12°, field of view (FOV) = 240 × 200 mm^2^, in-plane resolution = 0.83 × 0.83 mm^2^, slice thickness = 0.8 mm, and number of slices = 192. To reduce the acquisition time, a generalized auto-calibrating partially parallel acquisition with an acceleration factor of 2 in the right-left direction and elliptical sampling were used. T1-weighted images were also obtained from all subjects by using a magnetization prepared rapid gradient echo sequence with the following parameters, namely, TR = 2,530 ms, inversion time (TI) = 1,100 ms, TE = 2.98 ms, FOV = 256 × 256 mm^2^, matrix = 256 × 256, slice thickness = 1 mm, and number of slices = 192.

The susceptibility maps were reconstructed using the Morphology-Enabled Dipole Inversion with automatic uniform cerebrospinal fluid (CSF) zero reference (MEDI+0) algorithm (Liu et al., 2018). First, the field map was estimated by performing a one-dimensional temporal unwrapping of the phase on each voxel followed by weighted least-squares fit of the temporally unwrapped phases in each voxel over TE (Liu et al., 2013). Then, a magnitude image-guided spatial unwrapping algorithm was used to account for frequency aliasing on the field map (Cusack and Papadakis, 2002). Thirdly, the background field was removed using the projection onto dipole fields (PDF) method (Liu et al., 2011). Finally, the remaining tissue field was inverted to generate a susceptibility map using MEDI (Liu et al., 2012), and the susceptibility of ventricular CSF was used as zero reference (Straub et al., 2017).

**Figure 1:**
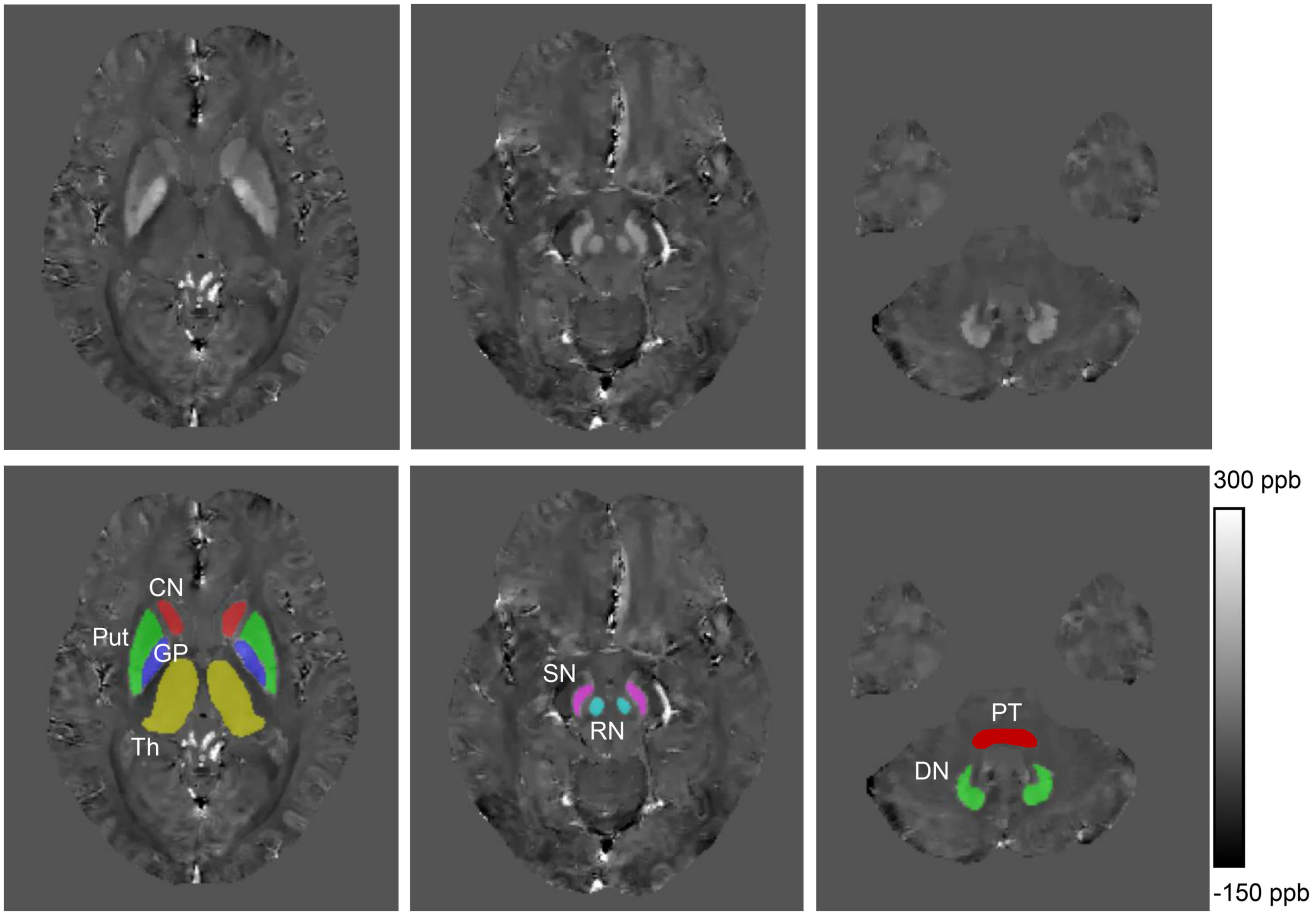
Example images showing regions of interest from a healthy control (41 years old, male). CN, head of the caudate nucleus; DN, dentate nucleus; GP, globus pallidus; Put, putamen; RN, red nucleus; SN, substantia nigra; Th, thalamus; PT, pontine tegmentum; ppb, part per billion.

Regions of interest (ROIs), including bilateral head of the caudate nuclei (CN), globus pallidus (GP), putamen (Put), thalamus (Th), substantia nigra (SN), red nuclei (RN), pontine tegmentum (PT), and dentate nuclei (DN), were manually segmented on susceptibility maps using ITK-SNAP (http://www.itk-snap.org). ROIs were drawn according to their anatomical boundaries on all sections where the outline of ROI was visible (Figure 1). The most inferior and most superior slice of ROIs were excluded to minimize partial volume effects. The ROIs were confirmed by a senior neurologist (XP. W.) with more than 30 years of experience. The volumes of ROIs were normalized for head size with skull-scaling factors, which were obtained from T1-weighted images using FSL's SIENAX tool (Smith et al., 2002). Susceptibility values of each ROI in the left and right hemispheres were averaged for further analysis. Total susceptibility was calculated using the formula total susceptibility = mean bulk susceptibility × the volume of ROI. This is derived from the definition of the mean bulk susceptibility, which represents the susceptibility value per volume.

### Statistical Analyses

A linear regression model was performed to determine group differences in mean bulk susceptibility, total susceptibility, and volumes of ROIs; age, gender, and disease duration were used as covariates to correct for their potential influence on these measurements. Distribution of the model residuals was visually evaluated using a P-P plot. Multiple comparisons between different ROIs were corrected by controlling the false discovery rate (FDR) at a level of 0.05 (Benjamini and Hochberg, 1995). Pairwise comparisons were performed among the three groups and *post-hoc* comparisons between groups were corrected by Bonferroni correction, and *p* < 0.017 (0.05/3) was considered significant. The area under the curve (AUC) was calculated by receiver operating characteristic curve (ROC) to analyze the diagnostic capability of total susceptibility and mean bulk susceptibility. The value of AUC varies from 0.5 to 1.0, the higher the value, the stronger the ability to distinguish between the two groups. Sensitivity and specificity of predictors in the diagnosis of WD were calculated by Youden index. The diagnostic accuracy of each region was also calculated. All statistical analyses were conducted using SPSS Statistics 22 (SPSS Inc, Chicago, IL, USA).

## Results

### Subjects' Characteristics

A total of 20 patients with neuro-WD (12 males, 8 females) and 10 hep-WD patients (7 males, 3 females) were enrolled in this study. The mean age of patients with neuro-WD and patients with hep-WD was 31.6 ± 7.69 (20–48) years and 28.9 ± 12.83 (11–45) years, respectively. The mean age of HCs was 29.48 ± 7.67 (18–43) years. The mean disease duration was 10.1 ± 6.7 (0.5–21) years for patients with neuro-WD and 7.79 ± 9.78 (0.5–32) years for patients with hep-WD. Disease duration was calculated from the time of symptom onset to the time of MRI scans. Detailed information about subject characteristics can be found in Table 1.

**Table 1:** Demographic and clinical characteristics of patients with WD and healthy controls.

	**Neuro-WD**	**Hep-WD**	**Healthy controls**
Gender (male/female)	12/8	7/3	12/13
Age (years)	31.6 (7.69)	28.9 (12.83)	29.48 (7.67)
Disease duration (years)	10.1 (6.7)	7.79 (9.78)	—
Treatment duration (years)	7.65 (5.91)	6.03 (9.19)	—
Kayser-Fleischer rings	19/20	6/10	—
Ceruloplasmin (< 0.15 g/L)	20/20	10/10	—
D-penicillamine/DMSA	4/16	3/7	—

*Standard deviations are in parentheses*.

*Normal reference range: serum ceruloplasmin, 0.2–0.45 g/L*.

*DMSA, dimercaptosuccinic acid; Hep-WD, hepatic form of Wilson's disease; N, number; neuro-WD, neurological form of Wilson's disease*.

### Mean Bulk Susceptibility

**Figure 2:**
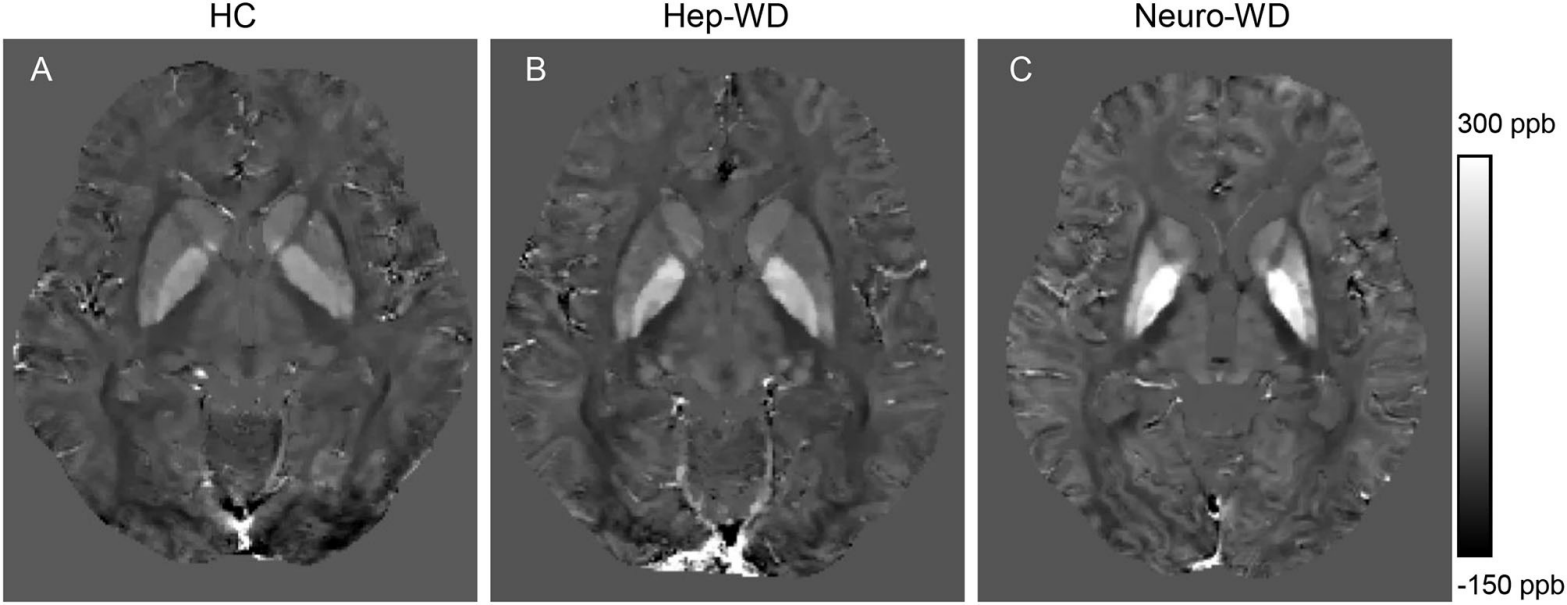
Brain susceptibility changes in patients with WD. **(A)** Normal susceptibility maps (28 years old, male). **(B)** Susceptibility maps from a patient with hep-WD (29 years old, male). **(C)** Susceptibility maps from a patient with neuro-WD (36 years old, male). ppb, part per billion.

**Figure 3:**
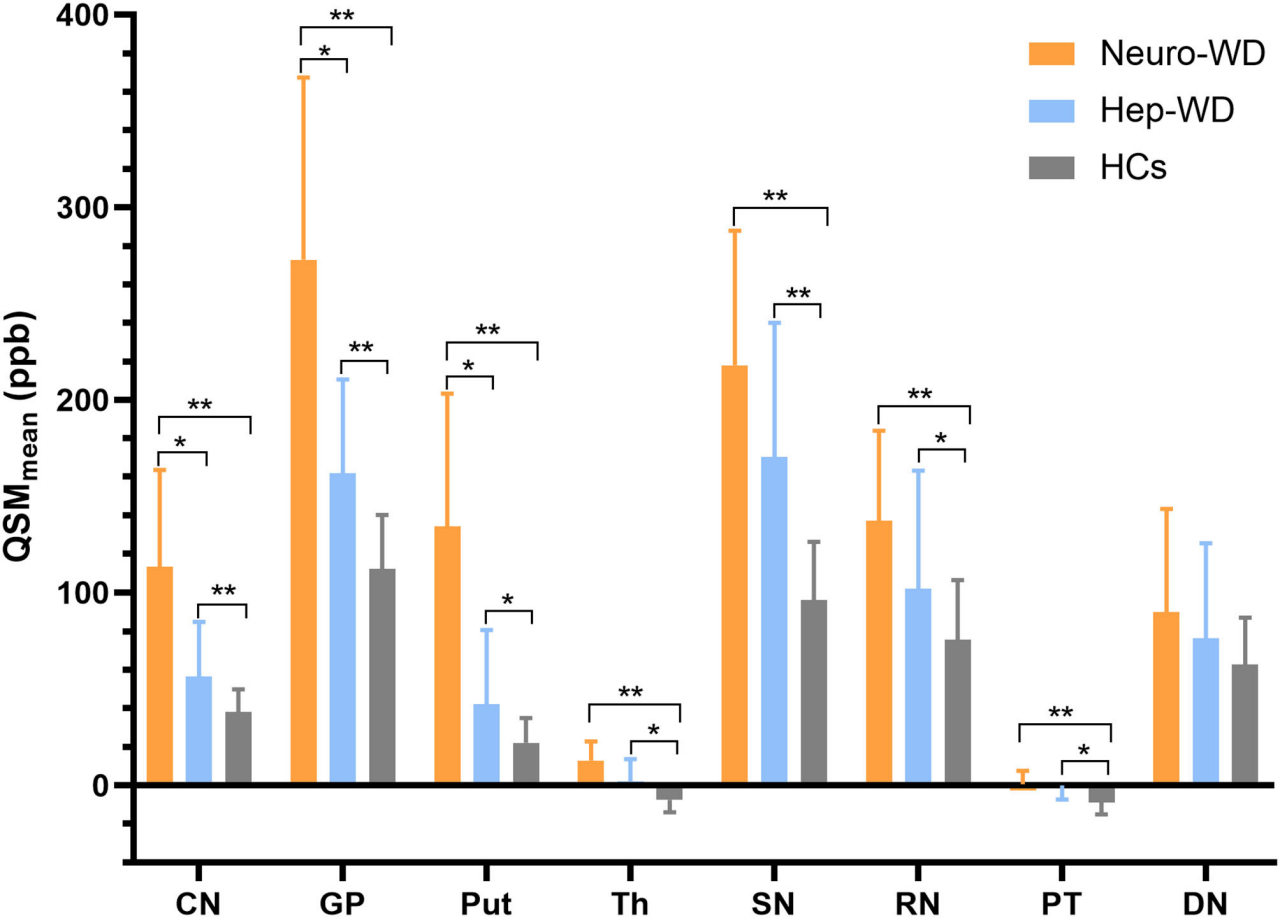
Comparison of mean bulk susceptibility values between different groups. Comparison between healthy control (HCs) and patients with WD: ^*^p < 0.017 and ^**^p < 0.001. Comparison between patients with neuro-WD and patients with hep-WD. Error bars indicate standard deviation. CN, head of the caudate nucleus; DN, dentate nucleus; GP, globus pallidus; Put, putamen; RN, red nucleus; SN, substantia nigra; Th, thalamus; PT, pontine tegmentum.

Compared with HCs (Figure 2A), increased mean bulk susceptibility was observed in the DGM nuclei of hep-WD patients (Figure 2B). Mean bulk susceptibility was higher in the head of the CN (*p* < 0.001), GP (*p* < 0.001), Put (*p* = 0.002), Th (*p* = 0.005), SN (*p* < 0.001), PT (*p* = 0.001), and RN (*p* = 0.006) of patients with hep-WD compared to HCs. However, mean bulk susceptibility was not significantly changed in the DN (*p* = 0.084) of patients with hep-WD. Significantly increased mean bulk susceptibility was observed in the CN, GP, Put, Th, SN, PT, and RN of patients with neuro-WD compared to HCs (*p* < 0.001). Slightly increased mean bulk susceptibility was also discovered in the DN of patients with neuro-WD, but it was not statistically significant (*p* = 0.032). Patients with neuro-WD tended to have higher mean bulk susceptibility values in the DGM nuclei than patients with hep-WD (Figure 2C). Patients with neuro-WD had higher mean bulk susceptibility in CN (*p* = 0.006), GP (*p* = 0.006), and Put (*p* = 0.006) compared to patients with hep-WD. Although mean bulk susceptibility of Th, SN, RN, PT, and DN was slightly higher in patients with neuro-WD than that in patients with hep-WD, the difference was not statistically significant (*p* > 0.017) (Figure 3, Supplementary Table 1).

### Volumes of Deep Gray Matter Nuclei

**Figure 4:**
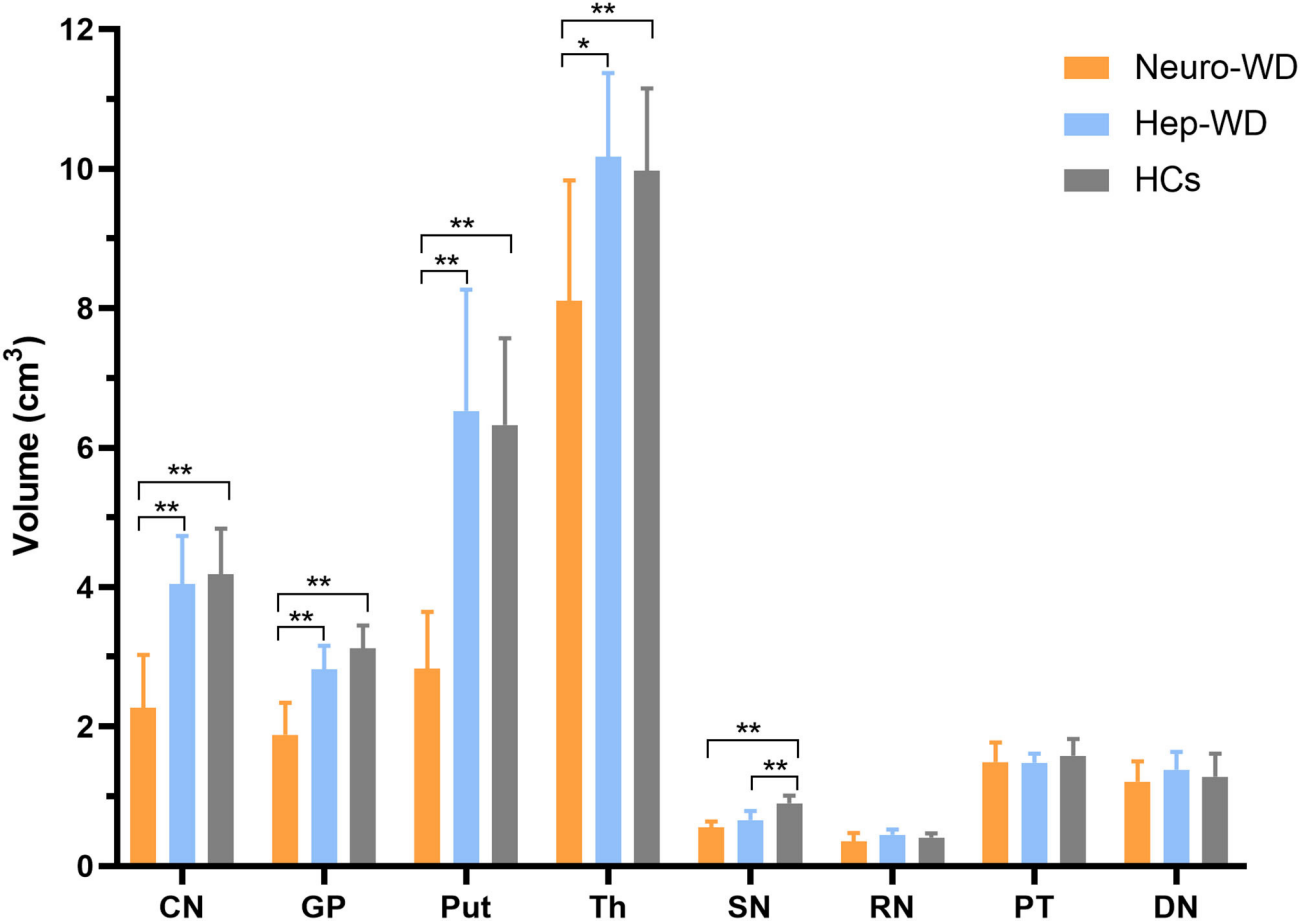
Comparison of volumes of deep gray matter nuclei between different groups. Comparison between healthy control (HCs) and patients with WD: ^*^p < 0.017 and ^**^p < 0.001. Comparison between patients with neuro-WD and patients with hep-WD. Error bars indicate standard deviation. CN, head of the caudate nucleus; DN, dentate nucleus; GP, globus pallidus; Put, putamen; RN, red nucleus; SN, substantia nigra; Th, thalamus; PT, pontine tegmentum.

Compared with HCs, patients with hep-WD had smaller volume in SN (*p* < 0.001), but the volumes of CN, GP, Put, Th, RN, PT, and DN were not significantly changed (*p* > 0.017). In patients with neuro-WD, volume reductions were observed in CN, GP, Put, Th, and SN compared to HCs (*p* < 0.001), but there was no significant difference in RN (*p* = 0.082), PT (*p* = 0.228), and DN (*p* = 0.411). Compared with patients with hep-WD, patients with neuro-WD had smaller volume in CN (*p* < 0.001), GP (*p* < 0.001), Put (*p* < 0.001), and Th (*p* = 0.003). No significant difference was found between patients with hep-WD and patients with neuro-WD in SN (*p* = 0.025), RN (*p* = 0.067), PT (*p* = 0.489), and DN (*p* = 0.121) (Figure 4, Supplementary Table 2).

### Total Susceptibility

**Figure 5:**
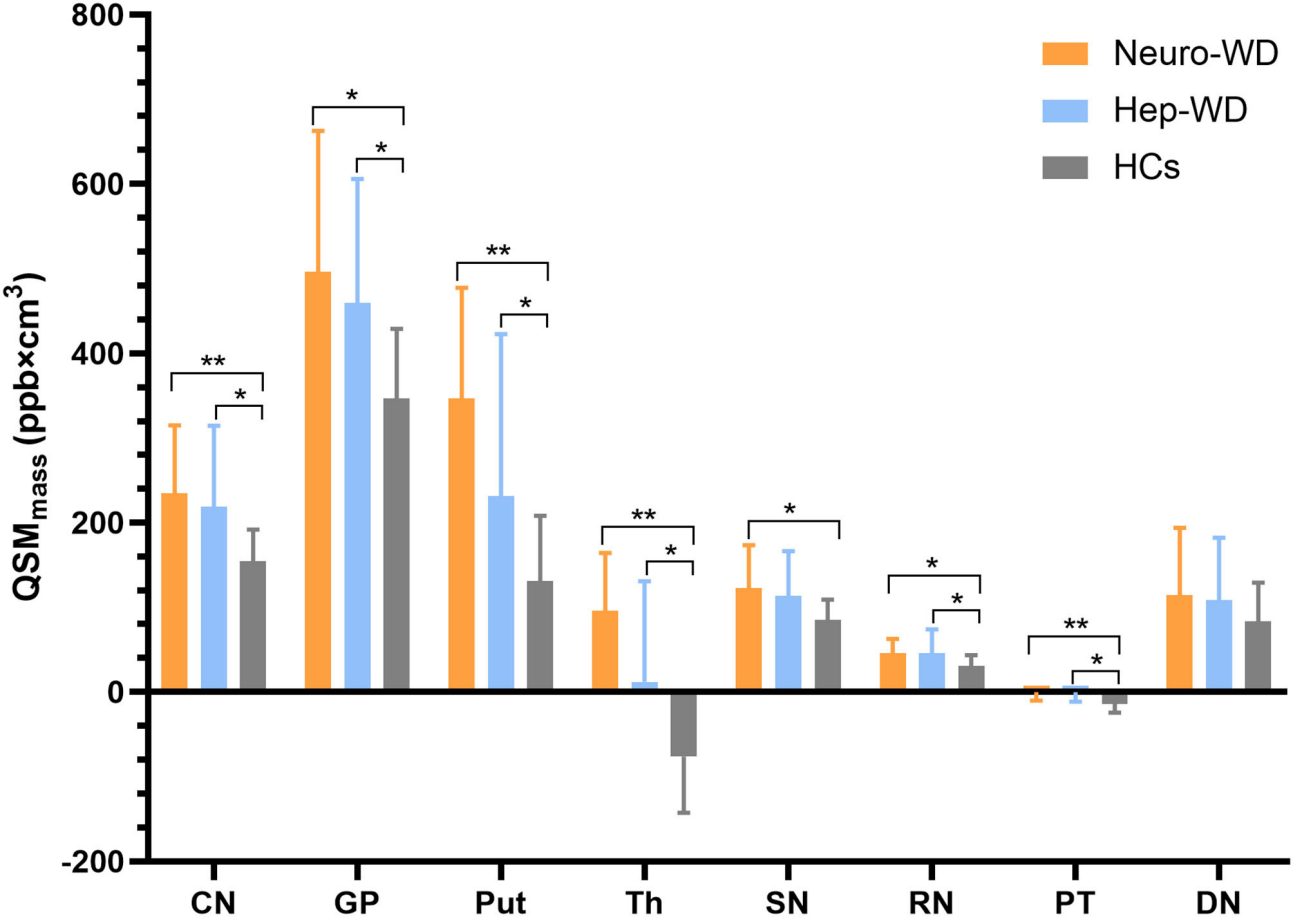
Comparison of total susceptibility values between different groups. Comparison between healthy control (HCs) and patients with WD: ^*^p < 0.017 and ^**^p < 0.001. Patients with neuro-WD and patients with hep-WD had similar total susceptibility in all examined deep gray matter nuclei. Error bars indicate standard deviation. CN, head of the caudate nucleus; DN, dentate nucleus; GP, globus pallidus; Put, putamen; RN, red nucleus; SN, substantia nigra; Th, thalamus; PT, pontine tegmentum.

Total susceptibility was increased in the CN (*p* = 0.001), GP (*p* = 0.003), Put (*p* = 0.001), Th (*p* = 0.001), PT (*p* = 0.002), and RN (*p* = 0.011) of patients with hep-WD compared to HCs. Total susceptibility was not significantly changed in the SN (*p* = 0.019) and DN (*p* = 0.087) of patients with hep-WD. Compared with HCs, patients with neuro-WD had higher total susceptibility in CN (*p* < 0.001), GP (*p* = 0.001), Put (*p* < 0.001), Th (*p* < 0.001), SN (*p* = 0.004), PT (*p* < 0.001), and RN (*p* = 0.003). Total susceptibility of DN was still not significantly changed in patients with neuro-WD (*p* = 0.138). Patients with neuro-WD and patients with hep-WD had similar total susceptibility in all examined ROIs (*p* > 0.017) (Figure 5, Supplementary Table 3).

### ROC Curve Analyses Between Patients With Neuro-WD and HCs

**Figure 6:**
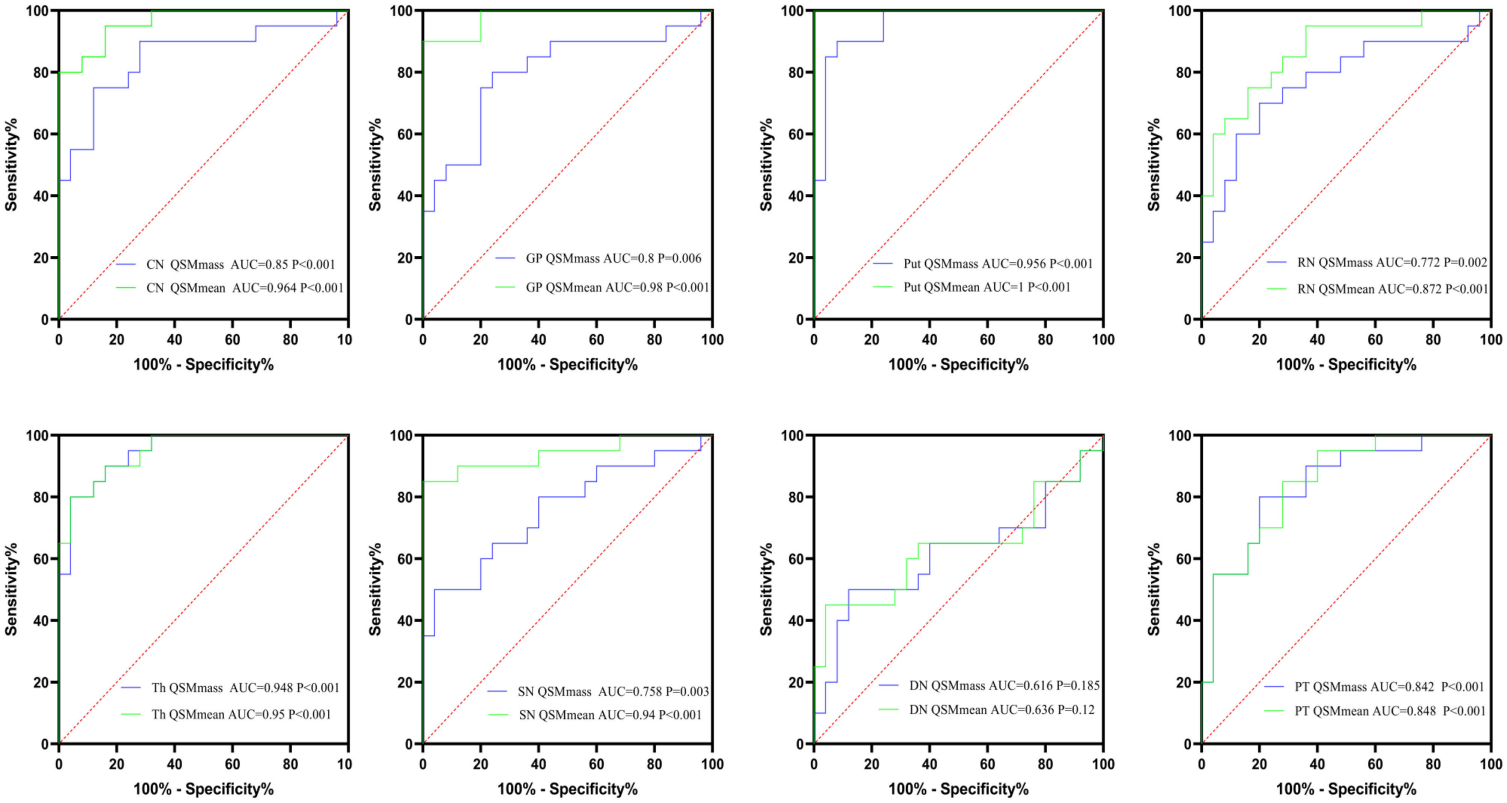
The results of ROC curve analyses of mean bulk susceptibility and total susceptibility between neuro-WD and HCs. CN, head of the caudate nucleus; DN, dentate nucleus; GP, globus pallidus; Put, putamen; RN, red nucleus; SN, substantia nigra; Th, thalamus; PT, pontine tegmentum.

AUCs for the mean bulk susceptibility of CN, GP, Put, Th, SN, RN, PT, and DN were 0.964 (*p* < 0.001), 0.98 (*p* < 0.001), 1 (*p* < 0.001), 0.95 (*p* < 0.001), 0.94 (*p* < 0.001), 0.872 (*p* < 0.001), 0.848 (*p* < 0.001), and 0.636 (*p* = 0.12), respectively, thus showing that the mean bulk susceptibility of Put was better than other regions in distinguishing patients with neuro-WD from HCs. For total susceptibility, AUCs for CN, GP, Put, Th, SN, RN, PT, and DN were 0.85 (*p* < 0.001), 0.8 (*p* = 0.001), 0.956 (*p* < 0.001), 0.948 (*p* < 0.001), 0.758 (*p* = 0.003), 0.772 (*p* = 0.002), 0.842 (*p* < 0.001), and 0.616 (*p* = 0.185), respectively, indicating that total susceptibility of Put was superior than other regions in classifying patients with neuro-WD. Basal ganglia nuclei (CN, Put, Th, and GP) showed better performance than brainstem nuclei (SN, RN) in both mean bulk susceptibility and total susceptibility for this study. Taken together, mean bulk susceptibility of Put provided the best diagnostic capability for neurological WD, AUC value was 1, and sensitivity and specificity were all equal to 1. The results of ROC curve analyses of mean bulk susceptibility and total susceptibility between neuro-WD and HCs are summarized in Figure 6, Supplementary Table 4.

### ROC Curve Analyses Between hep-WD and HCs

**Figure 7:**
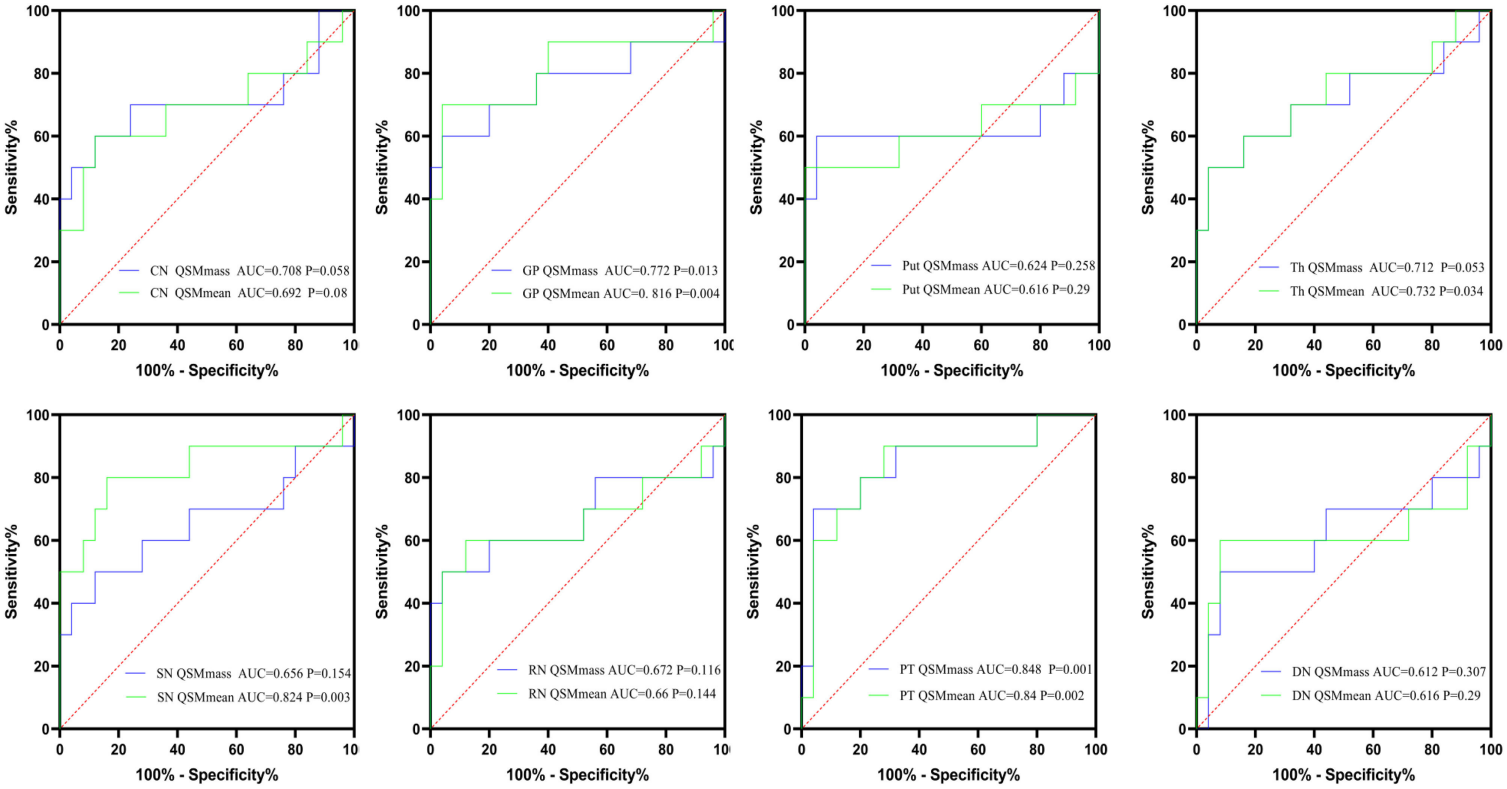
The results of ROC curve analyses of mean bulk susceptibility and total susceptibility between hep-WD and HCs. CN, head of the caudate nucleus; DN, dentate nucleus; GP, globus pallidus; Put, putamen; RN, red nucleus; SN, substantia nigra; Th, thalamus; PT, pontine tegmentum.

AUCs for the mean bulk susceptibility of CN, GP, Put, Th, SN, RN, PT, and DN were 0.692 (*p* = 0.08), 0.816 (*p* = 0.004), 0.616 (*p* = 0.29), 0.732 (*p* < 0.034), 0.824 (*p* = 0.003), 0.66 (*p* = 0.144), 0.84 (*p* = 0.002), and 0.616 (*p* = 0.29), respectively. For total susceptibility, AUCs for CN, GP, Put, Th, SN, RN, PT, and DN were 0.708 (*p* = 0.058), 0.772 (*p* = 0.013), 0.624 (*p* = 0.258), 0.712 (*p* = 0.053), 0.656 (*p* = 0.154), 0.672 (*p* = 0.116), 0.848 (*p* = 0.001), and 0.612 (*p* = 0.307), only GP and PT revealed statistically significant differences in ROC curve analyses. For patients with hep-WD, brainstem nuclei (SN, PT) revealed higher AUC values than basal ganglia nuclei (CN, GP, and Put). In general, total susceptibility of PT was more appropriate for the diagnosis of hep-WD than other regions with the highest AUC value (0.848) and diagnostic accuracy (88.57%). The results of ROC curve analyses of mean bulk susceptibility and total susceptibility between hep-WD and HCs are summarized in Figure 7, Supplementary Table 5.

## Discussion

In this study, significantly increased mean bulk susceptibility and total susceptibility were observed in multiple DGM nuclei of both patients with hep-WD and neuro-WD. We found that there was no difference in total susceptibility between the two groups of patients with WD in all examined ROIs. However, the mean bulk susceptibility of patients with neuro-WD in CN, GP, and Put was higher than those of patients with hep-WD. The result may be explained by the more significant atrophy of CN, GP, and Put in patients with neuro-WD compared with those in patients with hep-WD. The above results demonstrated that regional atrophy could affect the assessment of mean bulk susceptibility. In addition, the results of our study also revealed that mean bulk susceptibility of Put had excellent diagnostic capability for neuro-WD, and total susceptibility of PT provided the best diagnostic capability for hep-WD, suggesting that total susceptibility of DGM nuclei may be another useful imaging marker for the early diagnosis of WD.

In this study, we found significantly increased mean bulk susceptibility in all examined DGM nuclei except DN in patients with both neurological and hepatic WD. Recent MRI-histopathological studies identified intracellular ferritin and hemosiderin as the dominant source of the DGM susceptibility in healthy individuals (Langkammer et al., 2012; Hametner et al., 2018). WD is a copper-overload disease, and brain copper concentration is about 10-fold higher than in normal conditions (Poujois et al., 2017). Previous studies attributed the susceptibility changes in WD to copper accumulation or to copper and iron accumulation (Fritzsch et al., 2014; Doganay et al., 2018; Saracoglu et al., 2018; Dezortova et al., 2020). *In vitro* evidence suggests that Cu^2+^ compounds are slightly paramagnetic and Cu^+^ compounds are diamagnetic (Valdes Hernandez Mdel et al., 2012). However, the magnetic features of copper compounds *in vivo* are unclear. Only reduced copper (Cu^+^) can be transported into cells by copper transport receptor 1 (Ctr1), thus intracellular copper is likely to be transported and stored as a diamagnetic form (Scheiber et al., 2017). In addition to copper accumulation, increased iron concentration was also detected in the brains of patients with WD (Poujois et al., 2017). The majority of intracellular iron is stored as ferric iron (Fe^3+^) in ferritin, which is highly paramagnetic (Dlouhy and Outten, 2013). A recent histopathological study also reported that the R2^*^ values of basal ganglia were associated with iron concentration rather than copper concentration (Dusek et al., 2018). Considering the fact that the iron concentration is about three times higher than that of copper in the DGM nuclei of patients with WD and iron compounds show much stronger paramagnetism than copper deposits (Dusek et al., 2018), we inferred that the increased susceptibility in the DGM nuclei was mainly caused by the increased iron concentration. However, we cannot completely rule out the effect of copper deposition on susceptibility changes.

The mean bulk susceptibility of multiple DGM nuclei in patients with hep-WD also increased significantly, indicating that the concentration of paramagnetic metals increased in the early stage of WD (before the onset of neurological symptoms). The mean bulk susceptibility of CN, GP, and Put in patients with neuro-WD was higher than those in patients with hep-WD, suggesting that there was higher metal concentration in the striatum of patients with neuro-WD. The pathological changes of patients with WD may be a continuous process, if not treated or improper measures, copper and iron may continue to deposit in the brain. With the deposition of metals in the brain, especially in the striatum, patients with WD may show neurological symptoms gradually. Striatum is the most vulnerable region in patients with neuro-WD, and the lesions in striatum were related to the severity of neurological impairment (Poujois et al., 2017). The increased metal concentration in striatum of patients with neuro-WD may be associated with the neurological symptoms such as tremor, dystonia, and choreic movement in patients with neuro-WD.

The mean bulk susceptibility of DN in patients with WD was slightly higher than that in HCs, but it was not statistically significant. Thus, DN in the cerebellum seems to be little affected by metal accumulation compared with other DGM nuclei. It is still controversial whether iron accumulates in the DN of patients with WD. A study analyzed brain tissue samples from 12 patients with WD found higher iron levels in DN of patients with WD, and iron content in DN was positively correlated to the duration of disease (Litwin et al., 2013). In contrast, another postmortem study of nine patients with WD did not find higher iron concentrations in patients with WD compared to control subjects (Dusek et al., 2018).

However, the increased mean bulk susceptibility of the DGM nuclei only reflects the increased metal concentration. DGM nuclei atrophy is a common feature of patients with neuro-WD and may affect the assessment of metal concentration (Hernandez-Torres et al., 2019; Smolinski et al., 2019; Dusek et al., 2021). In this study, we observed significant DGM nuclei atrophy in the CN, GP, Put, Th, and SN of patients with neuro-WD. In contrast, DGM nuclei atrophy was less common in patients with hep-WD and was only observed in SN. It is unclear whether total metal content increases in the DGM nuclei of patients with WD, especially in patients with neuro-WD. To evaluate total metal content in the DGM nuclei, we calculated the sum of susceptibility values within a region, i.e., total susceptibility. In patients with neuro-WD, increased total susceptibility was observed in all examined DGM nuclei except DN. There was no difference in total susceptibility between patients with neurological and hepatic WD in all examined regions, and the higher mean bulk susceptibility of CN, GP, and Put in neuro-WD may be due to the smaller volumes of this region compared with hep-WD. Taken together, the results of our study suggested that measurement of mean bulk susceptibility in patients with WD may be affected by brain atrophy, and total susceptibility may be an additional metric for total metal content assessment account for the effects of tissue atrophy.

In this study, we also found that both mean bulk susceptibility and total susceptibility were increased in Th and PT, which contains large amount of white matter fiber tracts. Myelin in white matter is diamagnetic, and demyelination can increase the susceptibility of Th (Hametner et al., 2018). Demyelination is a common pathological feature of white matter in patients with WD. Demyelination of Th and white matter in patients with WD has also been reported by recent studies using diffusion tensor imaging (DTI) (Li et al., 2014; Dong et al., 2019). Increased magnetic susceptibility of Th and PT in patients with WD may be the combined results of metal deposition and demyelination. However, we still cannot determine whether metal deposition or demyelination is the predominant factor that leads to the increased magnetic susceptibility in Th and PT, which may need to be further confirmed by histopathology studies.

Our study also found that the increase in paramagnetic metal (copper and iron) concentrations in DMG may provide a basis for the diagnosis of WD. In the analysis of QSM for the diagnosis of neuro-WD, mean bulk susceptibility of Put has the strongest ability to distinguish patients with neuro-WD from HCs. The AUC for mean bulk susceptibility of Put was 1, and the sensitivity, specificity, as well as accuracy of diagnosis were all equal to 1; neuropathological studies also found that subjects with WD showed typical pathological changes with tissue destruction of Put being the most serious. The putamen showed atrophy and tissue changes in varying degrees, ranging from mild astrogliosis, neuron loss, and rarefaction to severe cavitation with obvious astrocyte astrogliosis (Dusek et al., 2017). Patients with more prominent pathological severity in the Put showed elevated iron concentration that was related to an increased number of iron-containing macrophages. The tissue damage, cell infiltration, as well as astrocyte proliferation of GP and CN were considerably less pronounced compared to the Put (Dusek et al., 2017). Theoretically, it is difficult for a detection method to achieve 100% diagnostic accuracy. It may be due to the small sample size of this study. If the sample size was large enough, the accuracy of mean bulk susceptibility in Put for the diagnosis of neuro-WD may decrease slightly. In addition, the mean bulk susceptibility of GP, CN, Th, and SN can also distinguish neuro-WD from HCs very well, and the AUC values were all > 0.9. In the analysis of using total susceptibility to diagnose neuro-WD, the results obtained were similar to those by using mean bulk susceptibility. Although the AUC values for total susceptibility of DGM nuclei were lower than that for mean bulk susceptibility, the total susceptibility of Put and Th can also well distinguish neuro-WD from HCs, and the AUC values for Put and Th were 0.956 and 0.948, respectively.

For the diagnosis of hep-WD, mean bulk susceptibility in GP, Th, SN, and PT showed statistically significant differences in ROC curve analyses between the HCs and hep-WD groups, and the AUC values for mean bulk susceptibility of PT, GP, SN, and Th were 0.84, 0.816, 0.824, and 0.732, respectively. The mean bulk susceptibility of PT has the highest AUC value while the diagnostic accuracy was only 77.14%. For the ROC analysis of total susceptibility, only GP and PT revealed statistically significant differences, AUC for GP and PT was 0.772 and 0.848, and diagnostic accuracy was 85.71 and 88.57%. Taken together, total susceptibility of PT was superior to other regions in the diagnosis of hep-WD with the highest AUC value and diagnostic accuracy. PT contains a large amount of white matter fiber tracts, and myelin damage is a common feature of brain white matter in WD. A study found that oligodendrocytes are highly susceptible to copper poisoning and the swelling of myelin sheath was the earliest sign of experimental copper poisoning (Vogel and Evans, 1961). Interestingly, quantitative analysis of DTI parameters showed the altered tissue microstructure, even in the normal-appearing thalamus and lobar white matter (Li et al., 2014; Dong et al., 2019). Dusek et al. also found the atrophy of white matter within the pons, mesencephalon, internal capsule, and adjacent lobes by using deformation and surface-based morphometry (Dusek et al., 2021). Combined with the previous results, we speculate that myelin damage may exist in the pontine tegmental area of hep-WD, and myelin damage may be one of the early pathological changes of WD brain. This hypothesis needs to be further confirmed by histopathological studies.

Some limitations of the study have to be mentioned. First, in some patients with neuro-WD, DGM nuclei atrophy and local high susceptibility make the boundaries of DGM nuclei unclear, which may affect the accuracy of DGM nuclei segmentation. Second, all included patients with WD have received de-copper treatment, which may affect brain susceptibility to some extent. Brain susceptibility in untreated patients may better reflect metal distribution in patients with WD. Thirdly, a study of 183 healthy volunteers found lower total subcortical brain iron levels selectively in women from midlife, compared to men and younger women, indicating that age and gender may affect brain iron concentration in healthy adults (Persson et al., 2015). In our study, although we used a linear regression model to correct for the potential influence of age, gender, and disease duration on brain susceptibility, we still cannot completely rule out their influence on brain susceptibility. Finally, the sample size of hep-WD group is relatively small. The results of our study need to be confirmed in other large independent cohorts.

In summary, significantly increased mean bulk susceptibility and total susceptibility were observed in the majority of deep gray matter nuclei of both patients with neuro-WD and patients with hep-WD. Brain atrophy may affect the calculation of mean bulk susceptibility, and total susceptibility should be an additional metric for metal content assessment. Both mean bulk susceptibility and total susceptibility can be used as an imaging marker for the diagnosis of WD.

## Data Availability Statement

The original contributions presented in the study are included in the article/Supplementary Material, further inquiries can be directed to the corresponding author/s.

## Ethics Statement

The studies involving human participants were reviewed and approved by the Medical Ethics Committee of Tongren Hospital, Shanghai Jiao Tong University School of Medicine. Written informed consent to participate in this study was provided by the participants' legal guardian/next of kin.

## Author Contributions

X-ZJ, X-ZY, and G-YL developed the conception and design of the study, collected clinical and imaging data, analyzed, and interpreted the data. J-LC, RW, L-LY, and S-YZ collected and processed imaging data. J-QL and X-PW developed the conception, design of the study, and revised the manuscript. X-ZJ wrote the first draft. All authors critically reviewed the manuscript. All authors contributed to the article and approved the submitted version.

## Funding

This study was supported by grants from the National Natural Science Foundation of China (81671103 and 8201101090) and Shanghai Jiao Tong University Medical School Innovative Research Plan 2015 (TM201520).

## Conflict of Interest

The authors declare that the research was conducted in the absence of any commercial or financial relationships that could be construed as a potential conflict of interest.

## Publisher's Note

All claims expressed in this article are solely those of the authors and do not necessarily represent those of their affiliated organizations, or those of the publisher, the editors and the reviewers. Any product that may be evaluated in this article, or claim that may be made by its manufacturer, is not guaranteed or endorsed by the publisher.
