# Supplementary material for: Increased Magnetic Susceptibility in the Deep Gray Matter Nuclei of Wilson's Disease: Have We Been Ignoring Atrophy?
Source: Front Neurosci. 2022 Jun 1;16:794375. doi: 10.3389/fnins.2022.794375 (PMC9198485; doi:10.3389/fnins.2022.794375)
Supplement: Supplementary file 1 [file Data_Sheet_1.docx]

## Supplementary Materials

**Table 1** Mean bulk susceptibility values of ROIs in WD patients and heathy controls

|  | Neuro-WD (ppb) | Hep-WD (ppb) | HCs (ppb) |
| --- | --- | --- | --- |
| CN | 113.56 (50.11) ** # | 56.33 (28.53) ** | 38 (11.71) |
| GP | 272.71 (94.71) ** # | 161.9 (48.82) ** | 112.23 (28.09) |
| Put | 134.19 (69.1) ** # | 42.02 (38.6) * | 21.94 (13) |
| Th | 12.86 (9.82) ** | 1.94 (11.65) * | -7.52 (6.57) |
| SN | 218.05 (69.8) ** | 170.45 (69.61) ** | 96.01 (30.26) |
| RN | 137.24 (46.73) ** | 102.07 (61.3) * | 75.54 (30.93) |
| PT | 0.50(6.94) ** | -0.01 (7.31) * | -8.80 (6.39) |
| DN | 89.79 (53.65) | 76.12 (49.58) | 62.78 (24.32) |

Comparison of mean bulk susceptibility values between different groups. Comparison between healthy control (HCs) and WD patients: * indicates *P* < 0.017 and ** indicates *P* < 0.001. Comparison between neuro-WD patients and hep-WD patients: # indicates *P* < 0.017. CN, head of the caudate nucleus; DN, dentate nucleus; GP, globus pallidus; Put, Putamen; RN, red nucleus; SN, substantia nigra; Th, thalamus; PT, pontine tegmentum. ppb, part per billion.

**Table 2** Volumes of DGM in WD patients and heathy controls

|  | Neuro-WD (cm^3^) | Hep-WD (cm^3^) | HCs (cm^3^) |
| --- | --- | --- | --- |
| CN | 2.271 (0.759) **## | 4.045 (0.688) | 4.187 (0.652) |
| GP | 1.882 (0.458) **## | 2.821 (0.339) | 3.118 (0.333) |
| Put | 2.826 (0.82) **## | 6.527 (1.737) | 6.324 (1.245) |
| Th | 8.106 (1.726) **# | 10.169 (1.203) | 9.97 (1.182) |
| SN | 0.554 (0.084) ** | 0.658 (0.131) ** | 0.9 (0.111) |
| RN | 0.352 (0.121) | 0.449 (0.075) | 0.406 (0.064) |
| PT | 1.486 (0.281) | 1.482 (0.133) | 1.584 (0.241) |
| DN | 1.209 (0.292) | 1.379 (0.258) | 1.28 (0.33) |

Comparison of volumes of deep gray matter nuclei between different groups. Comparison between healthy control (HCs) and WD patients: * indicates *P* < 0.017 and ** indicates *P* < 0.001. Comparison between neuro-WD patients and hep-WD patients: # indicates *P* < 0.017 and ## indicates *P* < 0.001. CN, head of the caudate nucleus; DN, dentate nucleus; GP, globus pallidus; Put, Putamen; RN, red nucleus; SN, substantia nigra; Th, thalamus; PT, pontine tegmentum.

**Table 3** Total susceptibility values of ROIs in WD patients and heathy controls

|  | Neuro-WD (ppb×cm^3^) | Hep-WD (ppb×cm^3^) | HCs (ppb×cm^3^) |
| --- | --- | --- | --- |
| CN | 234.88 (80.08) ** | 218.63 (95.89) * | 154.72 (37.25) |
| GP | 496.38 (166.47) * | 459.44 (146.43) * | 346.58 (82.6) |
| Put | 346.97 (130.39) ** | 231.11 (191.74) * | 131.05 (77.21) |
| Th | 95.94 (68.28) ** | 12.04 (118.74) * | − 76.12 (66.35) |
| SN | 122.44 (51.08) * | 113.29 (53.09) | 85.13 (24.14) |
| RN | 46.22 (16.65) * | 45.81 (28.51) * | 30.55 (13.12) |
| PT | -0.34 (10.25) ** | -0.39 (10.98) * | -14.09 (10.35) |
| DN | 114.09 (79.94) | 108.54 (73.64) | 83.52 (45.89) |

Comparison of total susceptibility values between different groups. Comparison between healthy control (HCs) and WD patients: * indicates *P* < 0.017 and ** indicates *P* < 0.001. Neuro-WD patients and hep-WD patients had similar total susceptibility in all examined deep gray matter nuclei. CN, head of the caudate nucleus; DN, dentate nucleus; GP, globus pallidus; Put, Putamen; RN, red nucleus; SN, substantia nigra; Th, thalamus; PT, pontine tegmentum. ppb, part per billion.

**Table 4** Results of the ROC curve analyses of total susceptibility and mean bulk susceptibility between HCs and neuro-WD patients

|  | ROI | AUC | P | SS | SP | CV | AC (%) |
| --- | --- | --- | --- | --- | --- | --- | --- |
| QSM_mass_ | CN | 0.85 | <0.001 | 0.75 | 0.88 | 188.69 ppb | 72.4 |
|  | GP | 0.8 | 0.001 | 0.8 | 0.76 | 381.60 ppb | 77.8 |
|  | Put | 0.956 | <0.001 | 0.9 | 0.92 | 219.51ppb | 91.11 |
|  | Th | 0.948 | <0.001 | 0.8 | 0.96 | 29.23ppb | 88.9 |
|  | SN | 0.758 | 0.003 | 0.5 | 0.96 | 121.32ppb | 64.89 |
|  | RN | 0.772 | 0.002 | 0.7 | 0.8 | 39.36ppb | 75.56 |
|  | PT | 0.842 | <0.001 | 0.8 | 0.8 | -6.44ppb | 80 |
|  | DN | 0.616 | 0.185 | 0.5 | 0.88 | 118.24ppb | 71.1 |
| QSM_mean_ | CN | 0.964 | <0.001 | 0.8 | 1 | 73.24 ppb | 91.11 |
|  | GP | 0.98 | <0.001 | 0.9 | 1 | 172.2 ppb | 95.56 |
|  | Put | 1 | <0.001 | 1 | 1 | 49.99 ppb | 100 |
|  | Th | 0.95 | <0.001 | 0.8 | 0.96 | 2.89 ppb | 88.89 |
|  | SN | 0.94 | <0.001 | 0.85 | 1 | 156.8 ppb | 93.33 |
|  | RN | 0.872 | <0.001 | 0.95 | 0.64 | 80.06 ppb | 77.78 |
|  | PT | 0.848 | <0.001 | 0.85 | 0.72 | -4.96 ppb | 77.78 |
|  | DN | 0.636 | 0.12 | 0.45 | 0.96 | 92.95 ppb | 73.33 |

QSM_mass_: total susceptibility; QSM_mean_: mean bulk susceptibility; AUC: area under the curve; SS: sensitivity; SP: specificity; CV: cutoff values; AC: diagnostic accuracy. HCs: healthy controls; CN, head of the caudate nucleus; DN, dentate nucleus; GP, globus pallidus; Put, Putamen; RN, red nucleus; SN, substantia nigra; Th, thalamus; PT, pontine tegmentum.

**Table 5** Results of the ROC curve analyses of total susceptibility and mean bulk susceptibility between HCs and hep-WD patients

|  | ROI | AUC | P | SS | SP | CV | AC (%) |
| --- | --- | --- | --- | --- | --- | --- | --- |
| QSM_mass_ | CN | 0.708 | 0.058 | 0.6 | 0.88 | 190.78 | 80 |
|  | GP | 0.772 | 0.013 | 0.6 | 0.96 | 508.04 | 85.71 |
|  | Put | 0.624 | 0.258 | 0.6 | 0.96 | 235.34 | 85.71 |
|  | Th | 0.712 | 0.053 | 0.5 | 0.96 | 47.89 | 82.86 |
|  | SN | 0.656 | 0.154 | 0.5 | 0.88 | 111 | 77.14 |
|  | RN | 0.672 | 0.116 | 0.5 | 0.96 | 56.19 | 82.86 |
|  | PT | 0.848 | 0.001 | 0.7 | 0.96 | -2.23 | 88.57 |
|  | DN | 0.612 | 0.307 | 0.5 | 0.92 | 135.83 | 80 |
| QSM_mean_ | CN | 0.692 | 0.08 | 0.6 | 0.88 | 50.69 ppb | 80 |
|  | GP | 0.816 | 0.004 | 0.7 | 0.96 | 164.46 ppb | 88.57 |
|  | Put | 0.616 | 0.29 | 0.5 | 1 | 52.38 ppb | 85.71 |
|  | Th | 0.732 | 0.034 | 0.5 | 0.96 | 4.3 ppb | 82.86 |
|  | SN | 0.824 | 0.003 | 0.8 | 0.84 | 119.12 ppb | 82.86 |
|  | RN | 0.66 | 0.144 | 0.6 | 0.88 | 110.68 ppb | 80 |
|  | PT | 0.84 | 0.002 | 0.9 | 0.72 | -4.8 ppb | 77.14 |
|  | DN | 0.616 | 0.29 | 0.6 | 0.92 | 88.63 ppb | 82.86 |

QSM_mass_: total susceptibility; QSM_mean_: mean bulk susceptibility; AUC: area under the curve; SS: sensitivity; SP: specificity; CV: cutoff values; AC: diagnostic accuracy. HCs: healthy controls; CN, head of the caudate nucleus; DN, dentate nucleus; GP, globus pallidus; Put, Putamen; RN, red nucleus; SN, substantia nigra; Th, thalamus; PT, pontine tegmentum.
